# Investigation of Relationships Between the Geospatial Distribution of Cancer Incidence and Estimated Pesticide Use in the U.S. West

**DOI:** 10.1029/2021GH000544

**Published:** 2022-05-01

**Authors:** Naveen Joseph, Catherine R. Propper, Madeline Goebel, Shantel Henry, Indrakshi Roy, Alan S. Kolok

**Affiliations:** ^1^ Idaho Water Resources Research Institute University of Idaho Moscow ID USA; ^2^ Department of Biological Sciences Northern Arizona University Flagstaff AZ USA; ^3^ Center for Health Equity Research Northern Arizona University Flagstaff AZ USA

**Keywords:** pediatric cancer, pesticides, fumigants, metam, multilevel model, modifiable areal unit problem

## Abstract

The objective of the study was to evaluate the potential geospatial relationship between agricultural pesticide use and two cancer metrics (pediatric cancer incidence and total cancer incidence) across each of the 11 contiguous states in the Western United States at state and county resolution. The pesticide usage data were collected from the U.S. Geological Survey Pesticide National Synthesis Project database, while cancer data for each state were compiled from the National Cancer Institute State Cancer Profiles. At the state spatial scale, this study identified a significant positive association between the total mass of fumigants and pediatric cancer incidence, and also between the mass of one fumigant in particular, metam, and total cancer incidence (*P*‐value < 0.05). At the county scale, the relationship of all cancer incidence to pesticide usage was evaluated using a multilevel model including pesticide mass and pesticide mass tertiles. Low pediatric cancer rates in many counties precluded this type of evaluation in association with pesticide usage. At the county scale, the multilevel model using fumigant mass, fumigant mass tertiles, county, and state predicted the total cancer incidence (*R*‐squared = 0.95, NSE = 0.91, and Sum of square of residuals [SSR] = 8.22). Moreover, this study identified significant associations between total fumigant mass, high and medium tertiles of fumigant mass, total pesticide mass, and high tertiles of pesticide mass relative to total cancer incidence across counties. Fumigant application rate was shown to be important relative to the incidence of total cancer and pediatric cancer, at both state and county scales.

## Key Points

- This study analyzed the geospatial relationship between cancer metrics and pesticide usage in the 11 western states of the United States
- A multilevel model was employed, which accurately estimated the cancer incidence at county spatial resolution
- Fumigants were found to be statistically significantly associated with cancer incidence at both state and county spatial scales

## 1 Introduction

In recent years, several studies have explored the geospatial distribution of cancer incidence across the United States at state (Siegel et al., 2018) and county (Boscoe & Johnson, 2014; Farazi et al., 2018) levels of resolution, reflecting a growing recognition of the connections between geography and health. While these studies found statistically significant differences among states and regions, they did not attempt to relate those findings to environmental factors. Recently, studies have begun to analyze the relationship of cancer to various environmental agents such as pesticides (Leonel et al., 2020; Park et al., 2020; Werder et al., 2020). However, most of these studies focus on specific environmental agents and their relationship to specific cancer types such as brain tumors (Carles et al., 2017; Van Maele‐Fabry et al., 2017), thyroid cancer (Deziel et al., 2021; Lerro et al., 2018), and breast cancer (Pullella & Kotsopoulos, 2020; Wei & Zhu, 2019). While relevant, the studies above have neglected the cumulative impact of multiple environmental agents on cancer incidence.

Recent studies have highlighted the significance of shifting from a single environmental agent analysis toward the cumulative assessment of environmental agents and their association with cancer incidence (Joseph & Kolok, 2022; Lobdell et al., 2014; Messer et al., 2014; Scott et al., 2019). For instance, the concept of environmental burden index (Scott et al., 2019), which includes variables associated with poverty, income, food access, and race (Parrish, 2010), has demonstrated a relationship between socioeconomic characteristics and cancer incidence. While the environmental burden index has been successfully implemented, it does not include specific information related to the chemical contamination of air or water.

Relative to cancer, one environmental factor that may be particularly explanatory is the nationwide application of agricultural pesticides. In the United States alone, more than 1 billion pounds of pesticides are applied in the agricultural sector annually, accounting for approximately 20% of global pesticide load each year and 16%–18% of global pesticide expenditures (Atwood & Paisley‐Jones, 2017). This usage puts both rural and urban populations near these agricultural regions at an increased probability of agrichemical exposure. To date, most research regarding the relationship between pesticides and human cancer incidence has analyzed state‐level cohorts or specific case studies, very often focusing on laborers within the agricultural industry (Cantor et al., 1992), their families (Alavanja et al., 2004; Colt & Blair, 1998; Feychting et al., 2001), and residential exposure to agricultural pesticides (Teysseire et al., 2020). Data from the U.S. Agricultural Health Study, a longitudinal study of cancer and other health outcomes in a cohort of Iowa and North Carolina agrichemical applicators and their spouses, have identified significant linkages between cancer incidences and several agricultural chemicals (Cocco, 2016). Alavanja et al. (2004) and Andreotti et al. (2018) found significant correlations between pesticide exposure and pancreatic and prostate cancers among pesticide applicators. Similarly, studies examining the relationship between agrichemical use and cancer incidences in California noted an increased risk of several types of cancer—prostate, liver, stomach, and cervical cancers—among agrichemical applicators when compared to nonfarming California populations (Mills & Shah, 2014; Mills et al., 2005). Supporting this, Pluth et al. (2019) conducted a meta‐analysis of the literature, including 74 research articles, and found that 53 pesticides are associated with at least one cancer type and that 19 cancer types are associated with at least one pesticide type.

While the association between pesticide exposure and incidence of cancer has been extensively evaluated in agricultural populations, it has been more difficult to extrapolate these associations to broader populations, particularly at regional and national levels. This is a curious omission, as climate, water availability, and soil type primarily determine agricultural land use, and by extension, determine the pesticide profile for different counties, states, and geographical regions. Consequently, an analysis comparing pesticide use to population‐level cancer incidence at a broad geographic scale may be particularly insightful.

The objective of this study was to evaluate the potential geospatial relationship between agricultural pesticide use and two cancer metrics (pediatric cancer incidence and total cancer incidence) across each of the 11 contiguous states in the Western United States at county and state spatial resolutions. The current study compiled the cancer incidence data sets from the National Cancer Institute databases and compared those incidence rates to pesticide mass data sets from the Pesticide National Synthesis Project (PNSP) to understand the relationship between cancer incidences and pesticide use.

## 2 Methods

### 2.1 Study Area

The study area includes the 11 westernmost states of the contiguous United States: Arizona, California, Colorado, Idaho, Montana, Nevada, New Mexico, Oregon, Utah, Washington, and Wyoming.

### 2.2 Pesticide Data

The annual pesticide usage estimates for the year 2017 were obtained for 10 of the 11 conterminous states in the Western United States from the U.S. Geological Survey Pesticide National Synthesis Project (USGS‐PNSP) database (https://water.usgs.gov/nawqa/pnsp/usage/maps/compound_listing.php). At the time of data retrieval, the 2017 pesticide data were not available in the PNSP database for California. Therefore, the data for California were directly sourced from the California Department of Pesticide Regulation Pesticide Use Reporting (CDPR‐PUR) database (https://www.cdpr.ca.gov/docs/pur/pur17rep/statewide_ai_2017.htm).

While the database contains pesticide use estimates for over 500 different pesticides, this study focused on the 25 most applied conventional pesticide active ingredients in the agricultural market sector from 2008 to 2012 (as per Atwood and Paisley‐Jones (2017)). Table 1 lists the 25 pesticides categorized by type of pesticides—herbicide, insecticide, fungicide, fumigant, and plant growth regulator.

**Table 1:** The Top 25 Pesticides Identified in the U.S.

Type	Pesticide
Herbicide	2,4‐D, acetochlor, atrazine, dicamba, glufosinate, glyphosate, metolachlor, metolachlor‐S, paraquat, pendimethalin, propanil, trifluralin
Insecticide	Acephate, chlorpyrifos
Fungicide	Chlorothalonil, copper hydroxide, hydrated lime, mancozeb
Fumigant	Chloropicrin, di‐chloropropene, metam sodium, metam potassium, methyl bromide
Plant growth regulator	Decan‐1‐ol, ethephon

*Note.* EPA 2008–2012 pesticide industry sales and usage.

The PNSP data set includes high and low estimates of pesticide use in kilograms at the county level for all recorded pesticides used in a state. But for this study, we relied exclusively on the high pesticide use estimates to obtain a more conservative estimate of pesticide use and because there was a strong association between high and low estimates (*R* = 0.82 and *P*‐value < 0.01). Pesticide use data within the database are obtained from individual farms, aggregated, and reported at the multicounty Crop Reporting District (CRD) level. County‐level harvested crop acreage data are obtained from the USDA Census of Agriculture to calculate the median use rate of pesticide use by crop for all crops in each CRD. This median pesticide‐by‐crop rate is then applied to the harvested acreage for each crop in each county to determine high and low levels of estimated pesticide use at the county level (as per Baker and Stone (2013); Thelin and Stone (2013)). In contrast, data from CDPR‐PUR are initially reported at the county level. Low and high estimates of pesticide use in California are therefore equivalent. Data obtained from CDPR‐PUR are expressed in pounds and were subsequently converted to kilograms to be merged with the PNSP pesticide data.

Of the 25 most applied agricultural pesticides identified in Atwood and Paisley‐Jones (2017), all but one (Decan‐1‐ol) were used in at least one of the 11 states in 2017. Furthermore, metam sodium and metam potassium were combined into a single variable, metam, and similarly, metolachlor and metolachlor‐S were combined into a single variable, metolachlor. Consequently, the final analysis included 22 pesticides rather than the initial 25 listed in Table 1.

**Figure 1:**
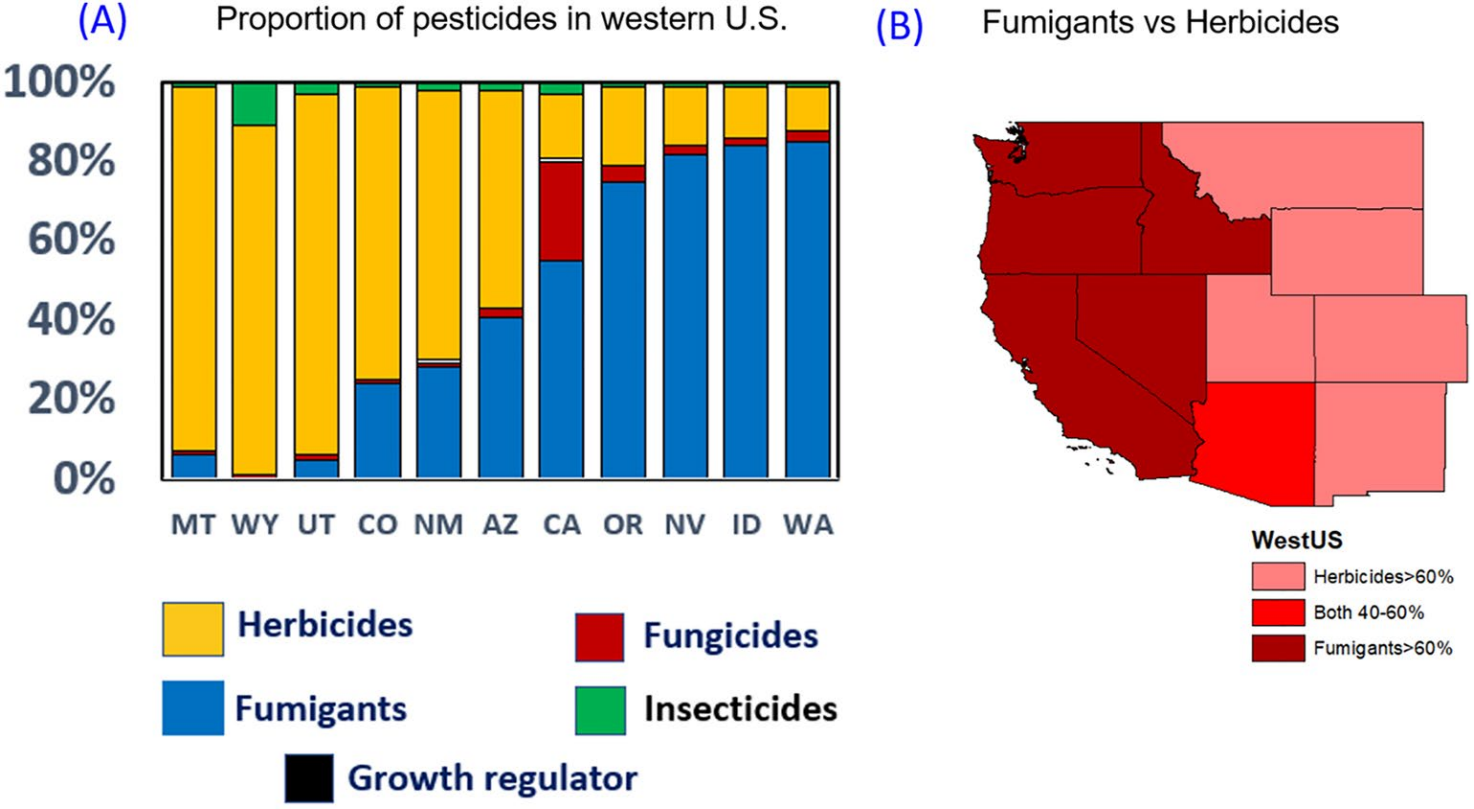
(a) Proportion of pesticide usage by state and (b) Spatial map of fumigant and herbicide proportion by state in the Western United States.

Of the 22 different pesticides that this project focused upon, most of the pesticides (by mass) used in each state tended to be either fumigants or herbicides. Herbicides dominated pesticide use in the eastern states of Wyoming, Colorado, Montana, New Mexico, and Utah, while fumigants dominated in the western states of Idaho, Washington, Oregon, California, and Nevada (Figure 1). In Arizona, herbicides and fumigants were applied at roughly equivalent rates. Indeed, when taken in combination, herbicides and fumigants comprised 71.5% of all the pesticides used in California (relative to the list of pesticides in Table 1) and no less than 88.8% of all of the pesticides used in the other 10 western states. The percent of applied pesticides that were fumigants ranged from a high of 85.2% in Washington to a low of less than 0.1% in Wyoming. Consistently, the percent of pesticides that were herbicides ranged from a high of 92% in New Mexico to a low of 11% in Washington. Among the fumigants, the most prevalently used fumigant was metam, and of the 22 pesticides evaluated in this study, the amount of metam used in Idaho, Washington, Oregon, and Nevada was higher than half of the total pesticide use. Likewise, among the herbicides, the most prevalently used herbicide was glyphosate, and of the 22 pesticides evaluated in this study, the amount of this single herbicide was higher than half of the total pesticide amount used in Montana and Wyoming.

### 2.3 Cancer Data

We obtained cancer data for each state individually from the National Cancer Institute State Cancer Profiles (https://statecancerprofiles.cancer.gov/incidencerates/). For each state, cancer data for the period 2012–2016 were aggregated at the county scale. We focused on age and population‐adjusted cancer incidence of all types of cancers and pediatric cancer (age < 20). Due to privacy concerns, counties with very low incidences of pediatric cancer were not reported, and hence county‐level pediatric cancer data were only available for 130 out of 414 counties. Therefore, the association of pediatric cancer to pesticides was only analyzed at the state scale. On the other hand, the association of all‐cancer incidence to pesticides was examined at both state and county resolutions.

### 2.4 Statistical Analysis at State Resolution

A series of linear regressions were run to analyze the associations between total cancer incidence, pediatric cancer incidence, and pesticide usage at state spatial scale. The pesticide usage data sets used include the mass of total pesticides, fumigants, herbicides, metam (dominant fumigant in the region), and glyphosate (most prevalent herbicide in the study area). The linear regressions were assumed to be statistically significant when *P*‐value < 0.05. The regressions were also evaluated using the coefficient of determination, R‐squared.

### 2.5 Statistical Analysis at County Resolution

**Figure 2:**
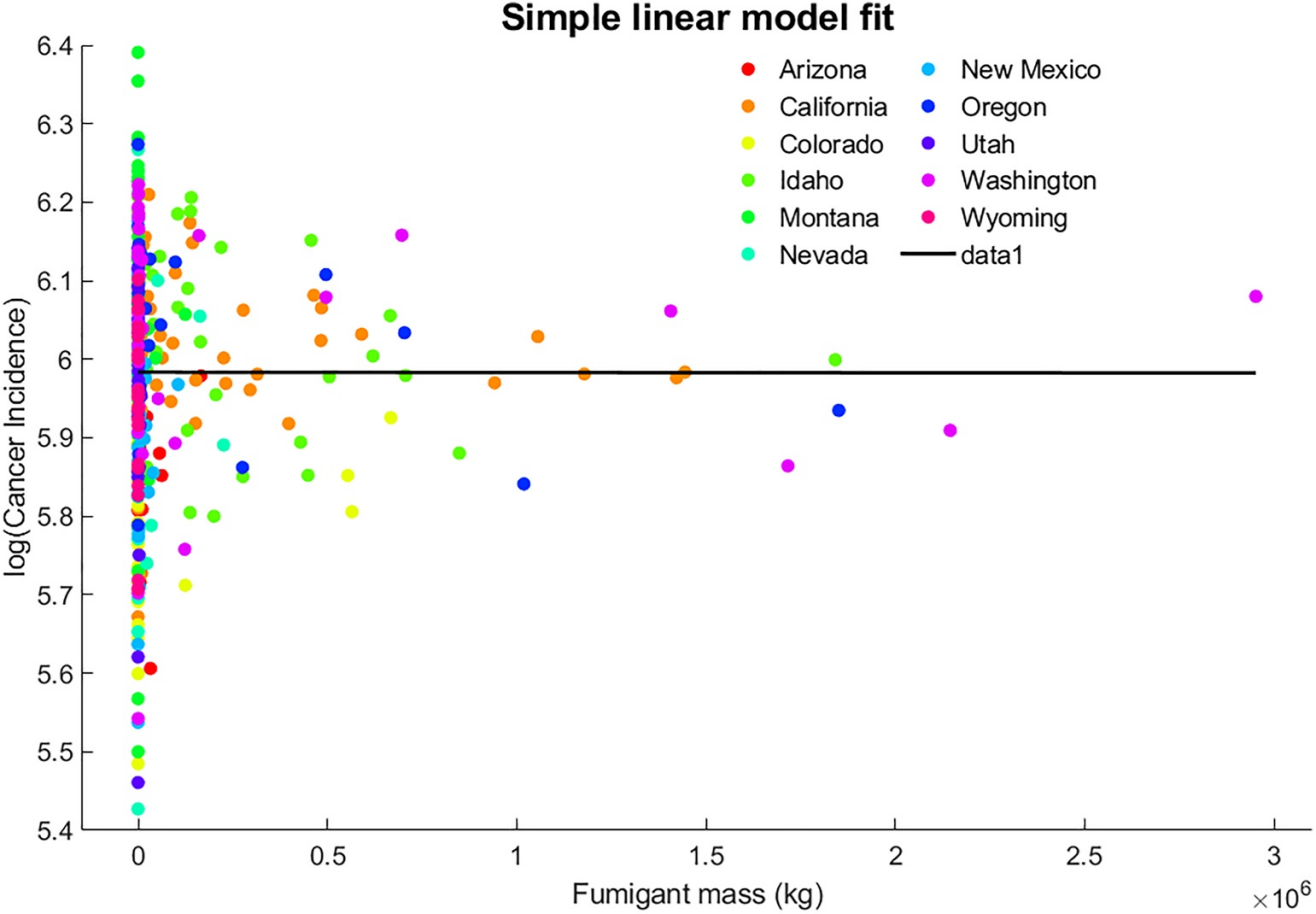
Cancer incidence against fumigant mass when a simple linear model is applied.

In addition to the linear regressions at the state level, the relationship of all‐cancer incidence to pesticide mass data sets at county resolution was also evaluated. The pesticide mass data sets considered at county resolution include total pesticide mass, fumigant mass, and herbicide mass. The pesticide types such as insecticides, fungicides, and plant growth regulators were not considered for individual analysis as those comprise less than 5%–10% in almost all the counties in the U.S. West. While using the data sets at county resolution, the linear regression method showed no specific trend. For instance, Figure 2 shows the all‐cancer incidence against fumigant mass at county scale for all the 11 states of the Western United States. The cancer incidence was log‐transformed for normalizing the data. Also, using this analysis methodology, there was no specific trend between the fumigant mass and cancer incidence at the county scale. Moreover, the distribution of cancer incidence against fumigant mass varied between the 11 states of the Western United States.

The difference in pattern between states was also similar when herbicide mass was used instead of fumigant mass. This is predominantly because the western region of the study area (Washington, Idaho, Oregon, Nevada, and California) is fumigant‐dominated. In contrast, the eastern part of the study area (Montana, Wyoming, Colorado, New Mexico, and Utah) is herbicide‐dominated. The state of Arizona has a similar loading of fumigants and herbicides. The usage of other types of pesticides was negligible in all 11 states, and hence, analysis concerning those types of pesticides was not performed.

To evaluate whether the state spatial resolution significantly impacted the model results, separate linear models were fitted for fumigant mass and all‐cancer incidence for each state. Figure S1 in Supporting Information S1 shows the intercept and slope for the linear model, and Figure S2 in Supporting Information S1 shows the residuals corresponding to each state. The individual confidence intervals do not overlap, suggesting the need to include state spatial scale into the model.

Further, separate linear models were fit for different fumigant mass classes, defined by the tertiles of fumigant mass at the county scale. Similar to Figure S1 in Supporting Information S1, the confidence intervals of the intercept and slope suggest the significance of also including the tertiles of fumigant mass (Figure S3 in Supporting Information S1) into the modeling framework.

#### 2.5.1 Multilevel Model Development

Three separate models were simulated for evaluating the relationship to all‐cancer incidence. In the first model, total pesticide mass, county, state, and pesticide mass tertile classes were selected as the variables for evaluating the relationship to all‐cancer incidence. The second model used fumigant mass and fumigant tertile classes instead of total pesticide mass, while the third model used herbicide mass and herbicide tertile classes. Multilevel modeling approach is used to control clustering of counties within states. MATLAB R2019a tool was used in this study for model simulation (MATLAB, 2019).

In the first model, the response variable selected was all‐cancer incidence. The independent variables selected were county, pesticide mass, and pesticide mass tertile classes. The state was considered as a random intercept. The distribution selected was normal distribution as the response variable, and the independent variables follow a normal distribution. The second and third multilevel models were simulated for (a) fumigants and (b) herbicides instead of total pesticides.

## 3 Results

### 3.1 Statistical Analysis Results at State Resolution

Two linear regressions were undertaken to predict the incidence rates of all cancers and pediatric cancers (separately) based on the total summed mass of all 22 pesticides. Across all states, regression analysis found a positive, albeit statistically weak association between the total summed mass of all 22 pesticides and total cancer incidence (400.69 + 1.834 × 10^−6^ pesticide mass, *R* ^2^ = 0.23, and *p* = 0.133) or pediatric cancer incidence (17.49 + 9.37 × 10^−8^ pesticide mass, *R* ^2^ = 0.33, and *p* = 0.063).

**Figure 3:**
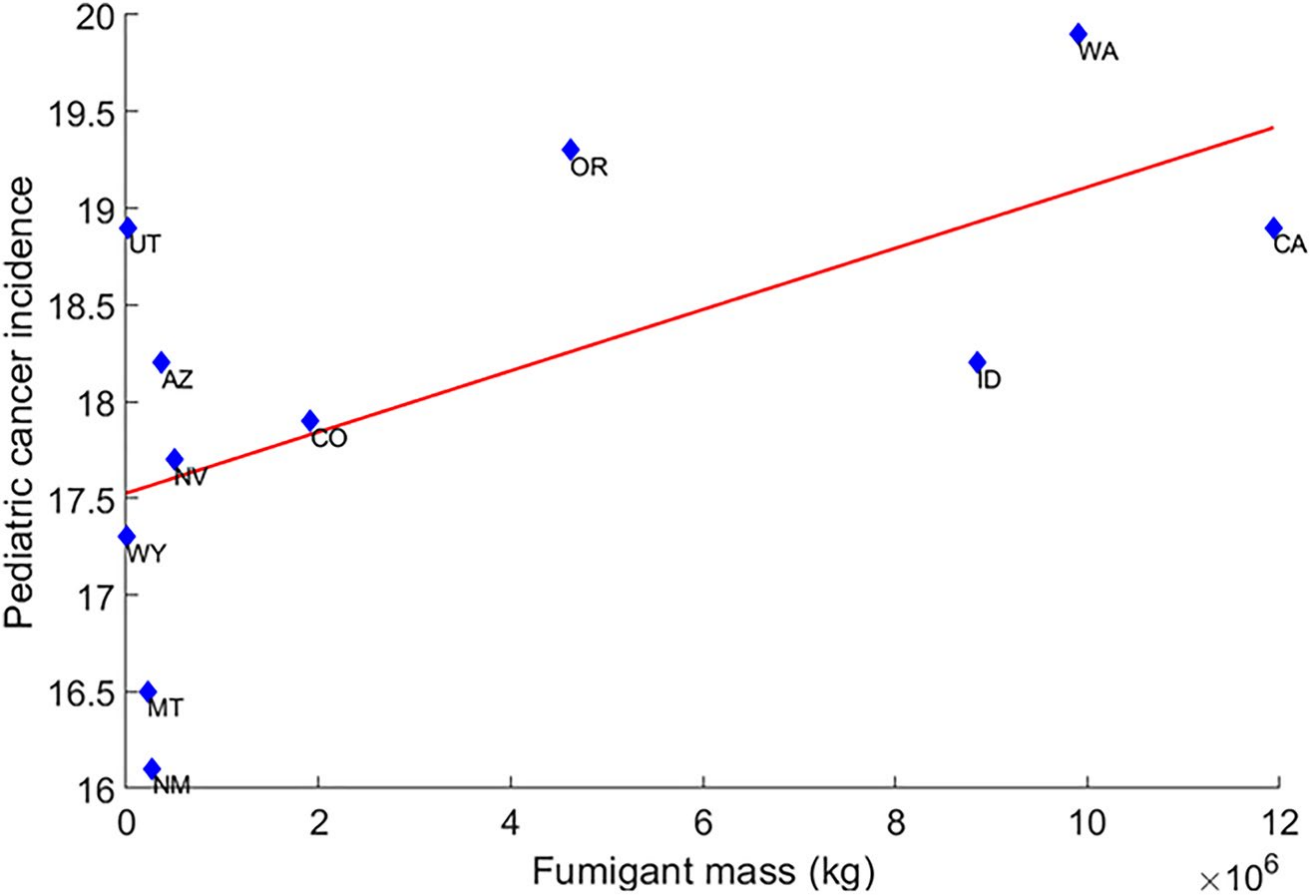
Pediatric cancer incidence against fumigant mass for the 11 western states of the United States.

When the total mass of herbicides in each state was regressed against total and pediatric cancer incidence, neither regression demonstrated an association (Total cancer incidence = 404.5 + 4.37 × 10^−6^ *Herbicide mass, *R* ^2^ = 0.09, and *p* = 0.37; Pediatric cancer incidence = 18.1–5.15 × 10^−9^ *Herbicide mass, *R* ^2^ = 0.001, and *p* = 0.98). In addition, the relationship between fumigant mass and all cancers did not show a strong association (Total cancer incidence = 401.6 + 2.77 × 10^−6^ *Fumigant mass, *R* ^2^ = 0.30, and *p* = 0.084). In contrast, a significant regression was found between the mass of fumigants applied in each state and the incidence of pediatric cancer across all states (Figure 3, Pediatric cancer incidence = 17.5 + 1.47 × 10^−7^ *Fumigant mass, *R* ^2^ = 0.45, and *p* = 0.022). The spatial autocorrelation of the residuals from the linear model was also selected as shown in Figure S4 in Supporting Information S1. The 99% confidence interval for the residual correlation was estimated as (−0.178, 0.078) which is close to zero. The linear model was selected for state‐wise analysis as the residuals were not highly correlated spatially.

**Figure 4:**
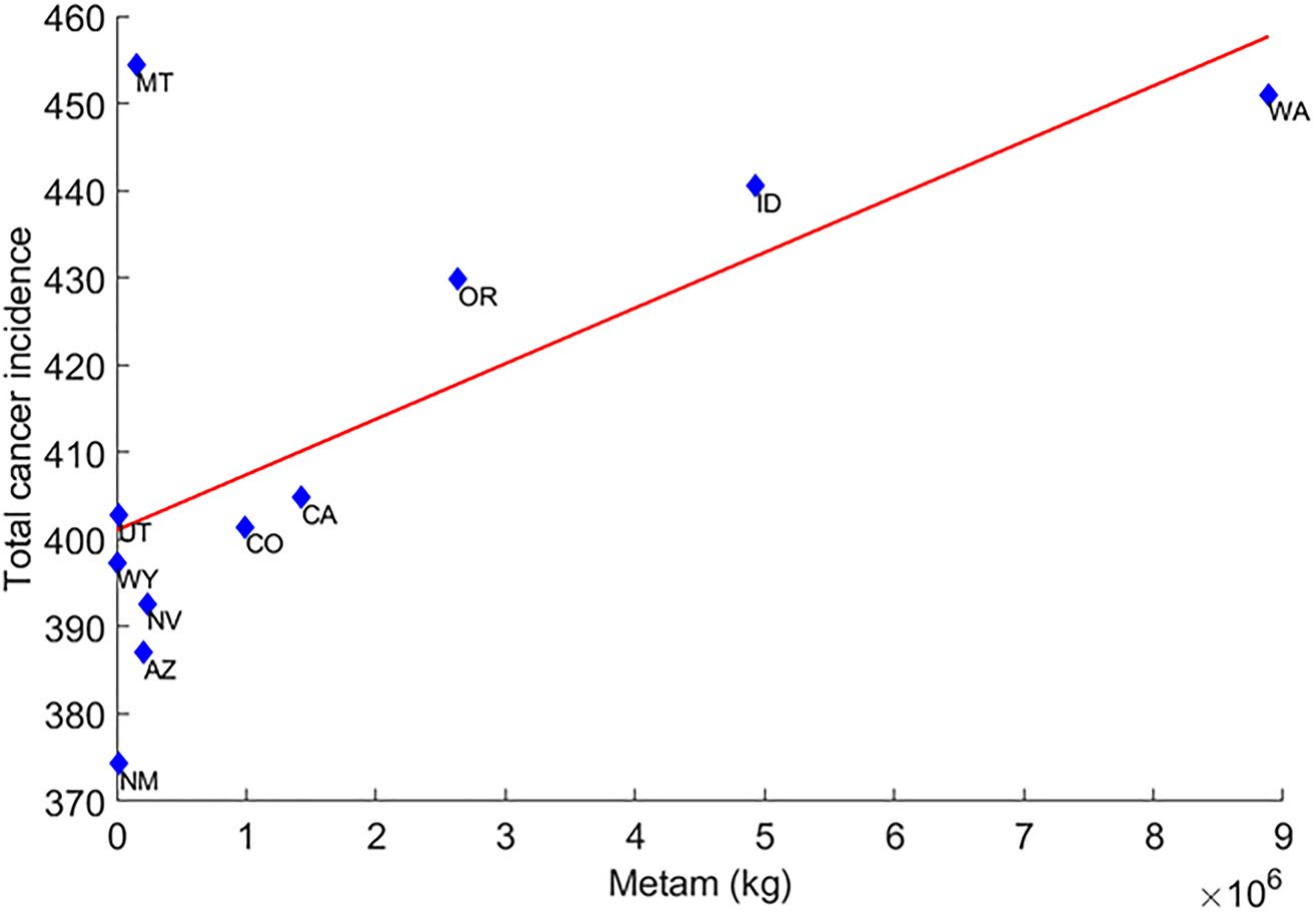
Total cancer incidence against metam for the 11 states of the western United States.

The relationships between the mass of glyphosate used in each state and either pediatric cancer or total cancer also showed no association (Pediatric cancer incidence = 18.3–2.13 × 10^−7^ *Glyphosate mass, *R* ^2^ = 0.03, and *p* = 0.61; Total cancer rate = 401.0 + 1.47 × 10^−5^ *Glyphosate mass, *R* ^2^ = 0.26, and *p* = 0.11). In contrast, the relationship between the mass of metam in each state and the incidence of pediatric cancers was weakly associated with pediatric cancer incidence (17.62 + 2.1 × 10^−7^ *metam, *R* ^2^ = 0.36, and *p* = 0.051); however, the relationship between metam mass and the incidence of total cancers showed a remarkable relationship (400.3 + 5.44 × 10^−6^ *metam, *R* ^2^ = 0.45, and *p* = 0.024, Figure 4).

### 3.2 Statistical Analysis Results at County Resolution

A multilevel model using normal distribution was adopted in this study. In the first model, when total pesticide mass and total pesticide mass tertile classes were used, total pesticide mass and high pesticide class (tertiles > 0.66) are statistically associated with all‐cancer incidence (*P*‐value < 0.01). In the second model, when fumigant mass and fumigant mass tertile classes were used, fumigant mass, high fumigant class (tertiles > 0.66), and medium fumigant class (0.33 < tertile < 0.66) were significantly associated with all‐cancer incidence (*P*‐value < 0.01). On the contrary, in the third model, when herbicide mass was used, only the low herbicide class showed a statistically significant relationship with all‐cancer incidences (*P*‐value < 0.01). This is primarily because the low herbicide class corresponds to the high fumigant class, which has a significant association with all‐cancer incidence, in most of the counties. In summary, total fumigant mass, medium and high fumigant classes, total pesticide mass, and high pesticide class were significantly positively associated with cancer incidence in the Western United States.

The fumigant mass model was selected for further analysis as the fumigants are found to strongly influence the geospatial distribution of all‐cancer incidence in the Western United States compared to total pesticides and herbicides. Table 2 shows the fixed‐effects coefficients of the developed model and the confidence interval of the estimates while using fumigant mass. Additionally, a type‐3 test for fixed effects (*F*‐test) was also performed (Keyes, 1999) as shown in Table S1 in Supporting Information S1. While *t*‐test can assess only one regression coefficient at a time, an *F*‐test can assess multiple coefficients simultaneously. It is important to note that both *F*‐test results (Table S1 in Supporting Information S1) and *t*‐test results (Table 2) are agreeing with each other. These results indicate that the fumigant mass and high and medium fumigant mass tertile classes significantly impact the response variable (all‐cancer incidence) at *P*‐value < 0.01 and *t*‐Stat > 2.5. This implies that higher application of fumigants is significantly associated with all‐cancer incidence in the Western United States at the county spatial scale.

**Table 2:** Fixed‐Effects Coefficients of the Model With Confidence Intervals Associating Fumigant Mass at the County Level With All‐Cancer Incidences

Name	Estimate	SE	t‐stat	*P*‐value	Lower C.I.	Upper C.I.
Intercept	6.0322	0.06711	89.888	4.47E‐69	5.8981	6.1663
Fumigant mass	5.38E‐08	1.8E‐08	2.9194	0.004836	1.7E‐08	9.06E‐08
Fumigant class—FM	0.075038	0.02623	2.8608	0.005702	0.022638	0.12744
Fumigant class—FH	0.07509	0.02019	3.7196	0.003978	0.03011	0.12006

*Note.* SE—Standard error, *t*‐stat—t‐statistic, C.I.—Confidence interval.

**Figure 5:**
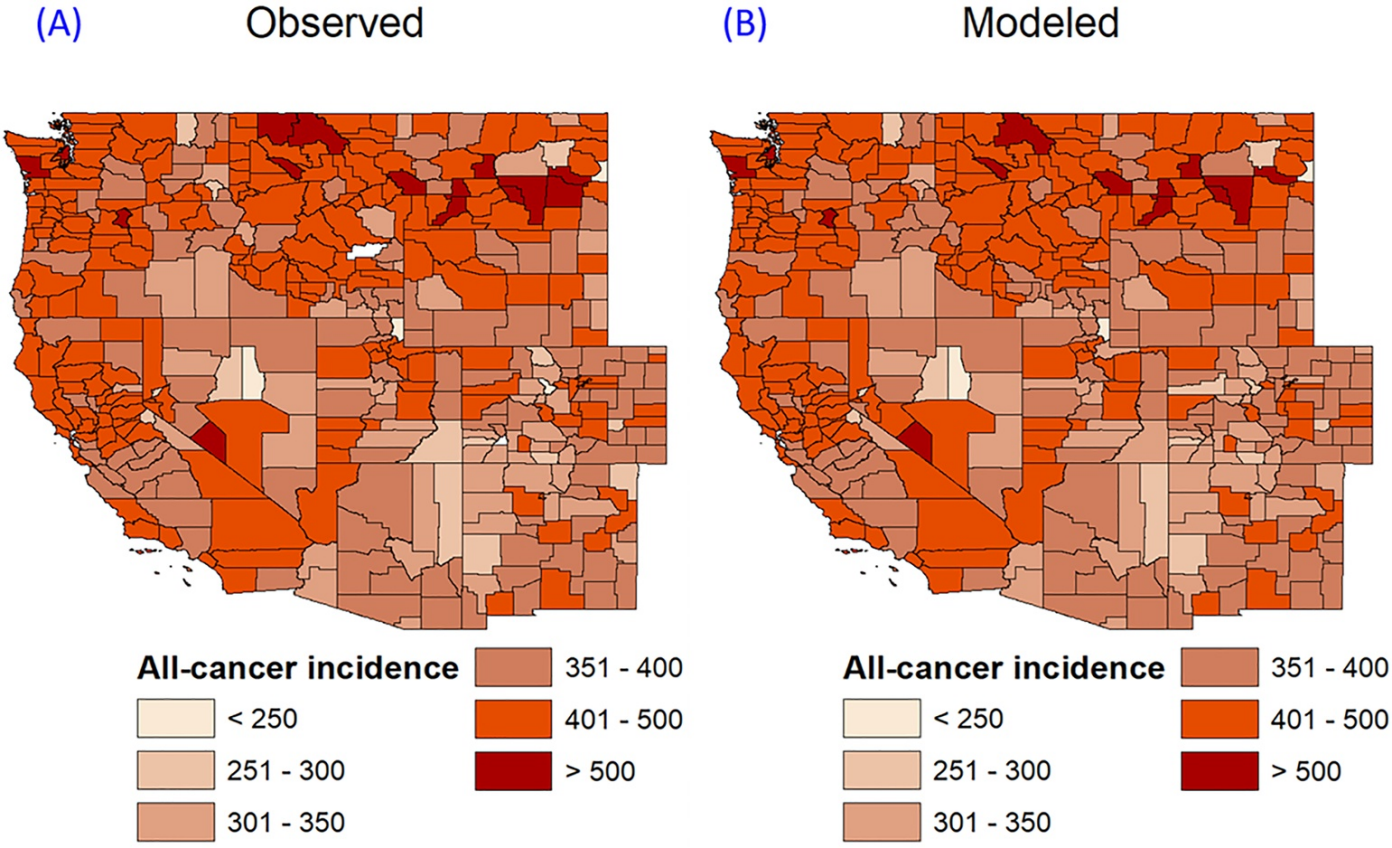
(a) Observed and (b) modeled all‐cancer incidence at county resolution for the 11 western states of the United States.

**Figure 6:**
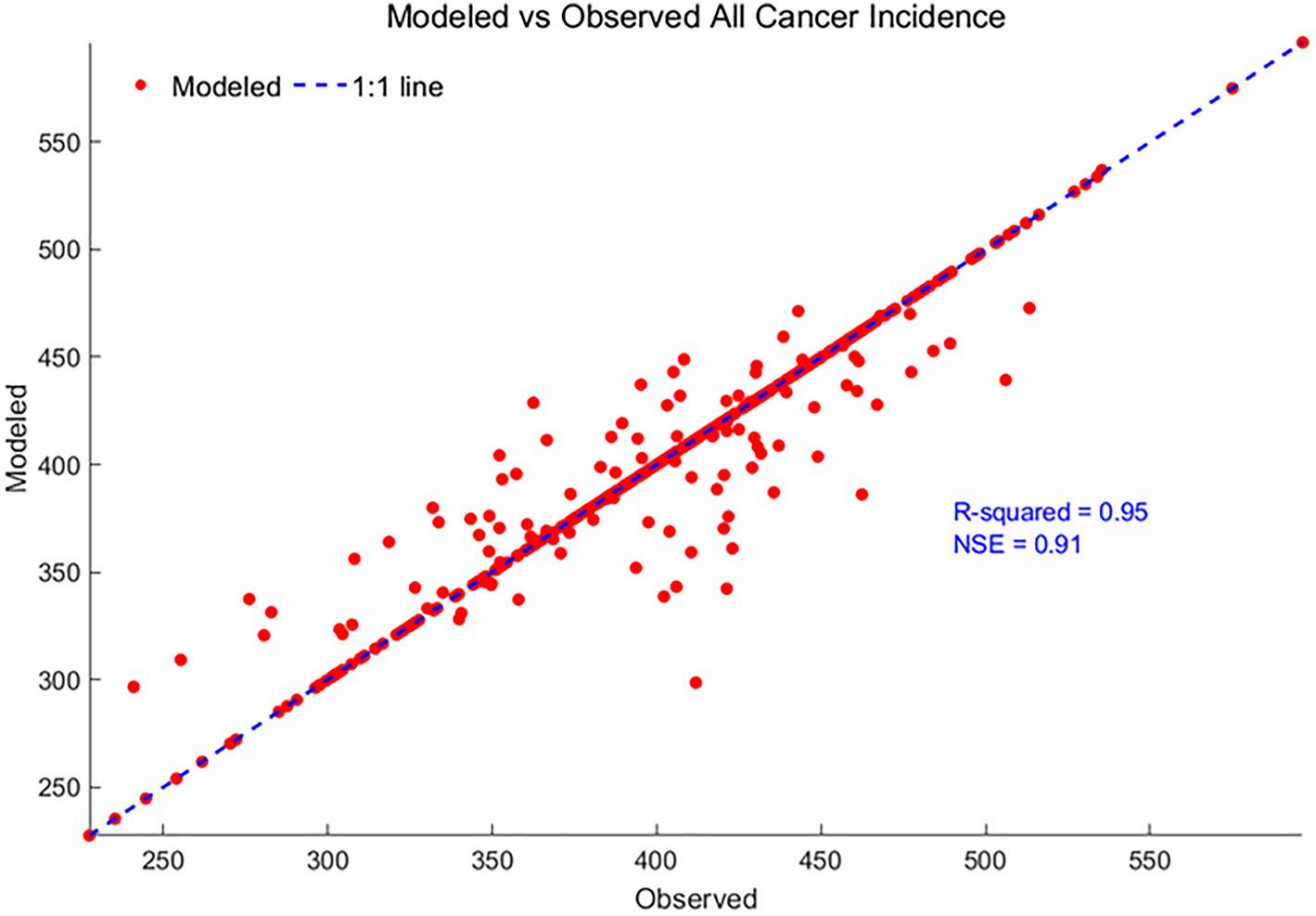
Modeled against observed all‐cancer incidence at county resolution in the Western United States.

Further, by using the developed multilevel model, the all‐cancer incidence at the county resolution was predicted as shown in Figures 5 and 6. Figures 5a and 5b shows the spatial map of the observed and modeled all‐cancer incidence using the fumigant mass and fumigant mass tertiles at the county resolution in the Western United States. The modeled results follow a similar pattern to the observed data across all the counties. Figure 6 shows the modeled against observed all‐cancer incidence with the 1:1 line added. The modeled results agree with the observed data accurately with R‐squared = 0.95 and NSE = 0.91. Moreover, the sum of the square of residuals (SSR) was estimated as 8.22, close to zero, further supporting the model selection. The spatial structure of the residuals at county scale was also plotted as shown in Figure S5 in Supporting Information S1. The darker blue color indicates the negative residual values, while the darker red color indicates the positive residual values, and white color indicates close to zero residual values. It is important to note that majority of the counties have close to zero residuals. While some counties have positive and negative residuals, the magnitude of residuals is not high and the spatial distribution of residuals are not clustered, further supporting the model selection.

## 4 Discussion

This study’s objective was to evaluate the potential geospatial relationship between agricultural pesticide use and two cancer metrics (pediatric cancer incidence and total cancer incidence) across each of the 11 contiguous states in the Western United States at the state and county spatial scales. At the state level of resolution, this study identified significant positive relationships between total mass of fumigants applied and pediatric cancer incidence and between the mass of metam (a fumigant) and total cancer incidence. At the county resolution scale, this study identified significant associations between total fumigant mass, high and medium tertiles of fumigant mass, total pesticide mass, and high tertiles of pesticide mass relative to total cancer incidence. Further, a multilevel model was developed using fumigant mass and fumigant mass tertiles across both states and counties, which accurately predicted total cancer incidence at the county scale. The fumigant application rate was shown to be important relative to total cancer and pediatric cancer incidence; moreover, this relationship was maintained regardless of whether the spatial resolution used in the analysis was conducted at the state or county level.

### 4.1 Significance of Consolidated Analysis of Pesticides

A consolidated analysis exploring the relationship between several types of pesticides and cancer incidence in the Western United States was performed in this study. This evaluation is relevant as it has become clear that a multifactorial approach to shift the current model of investigating a single environmental agent and its relationship to health outcomes, such as cancer, is critical to understand the complex nature of exposure to multiple chemicals (Boscoe et al., 2004; Hernández & Tsatsakis, 2017; Reynolds et al., 2004). One model for such an approach is the Environmental Burden Index (EBI), developed by Scott et al. (2019) that focused on socioeconomic and demographic variables such as poverty, income, food access, and race (Lantz & Pritchard, 2010), then it was used to evaluate associations with cancer mortality. Similar to the EBI, the United States Environmental Protection Agency (USEPA) has developed its own index, the Environmental Quality Index (EQI) (Lobdell et al., 2014), while Yale University developed a similar index, Environmental Performance Index (EPI) (Emerson et al., 2012) that focuses on several environmental domains. In contrast to EBIs, these latter indices are developed at a national level and have a coarser level of resolution. For instance, many of the environmental data sets used in these indices are collected at the state scale due to the scarcity of spatial and temporal data coverage at lower geographic levels of resolution (Messer et al., 2014). Consequently, they are of limited utility for public health officials who work at the state or local level. While these indices may be valuable with respect to modeling the incidence of different adverse health impacts, they are limited by the fact that they fail to incorporate variables associated with empirically measured environmental contamination. The approach we take here, allows for a more directed method for evaluating associations between environmental agents (chemical or societal) and health outcomes whether they are predicted to be positive or negative.

### 4.2 Modifiable Areal Unit Problem

Results from analysis at a single spatial unit may inadvertently be reliant upon the selected spatial unit, a concern known as the modifiable areal unit problem (Fotheringham & Wong, 1991; Openshaw, 1984). In other words, the results that might be produced when comparing adverse health outcomes on a state‐to‐state scale may not be consistent with the results obtained when the same comparisons are made on a county‐to‐county scale. Although the selection of scale depends to a large extent on data availability (S.‐I. Lee et al., 2019), analyses conducted at different spatial scales often produce statistically different results (Manley et al., 2006; Tuson et al., 2020; Wang & Di, 2020). For instance, several environmental epidemiological studies have quantified the impact of the areal unit problem in incidence rate mapping (Nakaya, 2000) by quantifying associations between nitrite and respiratory health analysis (Parenteau & Sawada, 2011), geospatial mapping of cerebrovascular diseases (Ayubi & Safiri, 2018), air pollution and health effects (D. Lee et al., 2020), and geospatial analysis of environmental factors and COVID‐19 death cases (Wang & Di, 2020). These studies infer that when incidence rates are mapped on smaller spatial units, they could be unreliable, and while mapped on larger spatial units, they may hide important geospatial variation (Nakaya, 2000; Nelson & Brewer, 2017). Recent studies such as Hennerdal and Nielsen (2017) have used a multiscale approach to overcome this spatial aggregation problem. Therefore, to overcome the statistical bias of modifiable areal unit problem, the current study approached the multivariable problem in the Western United States by conducting analyses at county and state spatial scales. The results obtained were consistent at both spatial scales, suggesting that the association between pesticides, specifically fumigants, and cancer incidence is worthy of attention.

### 4.3 Pesticides and Carcinogenicity at Different Geospatial Scales

The relationship between pesticides and carcinogenicity is one in which most of the epidemiological studies focus on agricultural workers and the potential cancer risks that come along with occupational exposure (Cocco, 2016; Mills & Shah, 2014). Fewer studies focus on the family members of agricultural workers (Daniels et al., 1997) and still fewer focus on individuals that are not members of these families (Deziel et al., 2015). Consequently, the geospatial orientation of most of these studies is at the level between local and statewide in agricultural states such as California (Mills & Shah, 2014; Mills et al., 2005; Park et al., 2020; Reynolds et al., 2004). These studies focused on the relationship between pesticides and carcinogenicity in agricultural workers as the association between a contaminant source and an exposure is most evidently a result of the geophysical exposure risk (Nuckols et al., 2004; Sahar et al., 2019). However, to the members of the public who are not applying pesticides, multiple exposure routes are possible depending on the location of their residence in relation to the pesticide applications (Alavanja et al., 2013).

By utilizing the Geographical Information System (GIS) in environmental epidemiology, studies have now been able to understand the association between environmental agents and health outcomes more efficiently (Meliker et al., 2005; Musa et al., 2013; Nuckols et al., 2004; Poulstrup & Hansen, 2004; Zhang & Zhang, 1997). These include, but are not limited to, geolocating the study population based on proximity to a contaminant source (Bell et al., 2001; Comba et al., 2003; Langholz et al., 2002), combining environmental monitoring data into adverse health outcomes (Floret et al., 2003; Reynolds et al., 2003), and GIS mapping to identify locations of high pesticide exposure regions so as to identify populations sensitive to health disparities (Christakos & Serre, 2000; Nuckols et al., 2004).

Although the geospatial distribution of pesticides is entirely different in the eastern and western regions of the study area, this study identified that the areas where a relatively larger mass of pesticides is applied are potentially more prone to increased cancer incidence than other counties. At the county level of resolution, either within a state, regional or national level analysis, the geophysical plausibility of pesticide exposure undoubtedly is diminished; however, the effective sample size increases. This study, though correlative, suggests that evaluating the links between pesticide usage and population level cancer outcomes may allow for determining research directions to determine whether specific pesticides are posing increased cancer risks for individuals. This result further supports the inferences from recent studies such as Pluth et al. (2019), who performed a meta‐analysis of 74 research articles on the association between pesticides and cancer, which identified that 64 among the 74 studies show a strong association between pesticide and cancer.

While the amount of pesticide used may vary slightly from year to year, it is our contention that the rank order of pesticide use, on a county‐by‐county basis across the Western United States does not appreciably change from 1 year to the next. Part of the reason for this is that there are counties where agriculture is chronically practiced, year in and year out, while there are other counties that are more closely focused upon forestry practices or urban/suburban human habitation. To support this claim, the Spearman rank of metam (most prominent pesticide) for the year 2017 against Spearman rank of metam for the year 2012 was also plotted as shown in Figure S6 in Supporting Information S1. The Spearman Rank Correlation between the use of metam in 2012 and 2017 for 194 counties in the 11 western states was extremely high (*R* = 0.95). Although the pesticide usage changes from 1 year to another, the rank order of counties is comparatively stable and as such, is highly useful relative to the generation of an environmental index or pesticide burden. This study did not perform an exposure‐based assessment, rather correlated the mass of pesticides applied in the counties of 11 western states (kg/county/area/year) to cancer metrics. While this study overcame the limitations of recent studies such as Scott et al. (2019) who used socioeconomic variables by using potential carcinogenic pesticides in the analysis, exposure routes were neglected. The future scope of this study is to overcome these limitations and collect additional temporal data sets on pesticides and cancer metrics to perform exposure‐based assessments.

### 4.4 Associations Between Fumigants and Cancer Incidence

This study identified a strong positive association between cancer incidence and fumigant mass and no association to herbicide mass. Although there are epidemiological studies analyzing the association between pesticide mass and cancer incidence within limited populations or scales, very few studies have focused on fumigant mass and cancer incidence. In a study investigating the linkages between pesticides and childhood acute lymphoblastic leukemia, Rull et al. (2009) found that children living close to agricultural fields in California had an increased cancer risk from exposure to fumigants. Other studies have found links between methyl bromide use and non‐Hodgkin’s lymphoma (Alavanja et al., 2014; Chiu et al., 2006) and stomach cancer (Barry et al., 2012). In contrast, other industry‐supported studies suggest that the fumigant 1,3‐dichloropropene, is applied at a level that should limit its risk of carcinogenicity (Driver et al., 2016; Yan et al., 2020). Similarly, there was also no association found between fumigant use and breast cancer in the wives of pesticide applicators (Werder et al., 2020), nor in glioma in the upper Midwest of the United States (Ruder et al., 2004). These epidemiology studies are largely limited in geospatial scale and population numbers. Furthermore, metam was not listed as one of the fumigants investigated in any of these risk evaluations.

Our study is one of the first to use publicly available databases, such as USGS‐PNSP, to identify associations between fumigants and cancer incidence at multiple spatial scales. Previous studies focused on pesticides and its association to cancer incidences using approaches such as parental interview in 35 California counties (Gunier et al., 2017) and surveys from pesticide applicators in North Carolina and Iowa (Alavanja et al., 2014; Andreotti et al., 2018). While relevant, one of the limitations of such studies is the lack of statistical power to investigate associations with individual pesticide, which was overcome by this study to an extent.

Previous studies focusing on linkages between pesticides and cancer incidence have used multilevel linear mixed effects models (Linhart et al., 2019; Shearer et al., 2019), and similar statistical techniques such as Cox proportional hazards regression (Werder et al., 2020), and weighted quantile sum regression (Zhang et al., 2019). Similar to that, this study used a multilevel model to investigate associations between pesticides and cancer metrics. While majority of the previous studies focused on specific regions or locations within a state, this is one of the first study to analyze the geospatial distribution of cancer incidence with respect to pesticides in the Western United States.

#### 4.4.1 Metam and Links to Cancer

Metam sodium and metam potassium are dithiocarbamate salts and are among the most used fumigants in the United States. They have been used since 1975 as soil fumigants to control a wide variety of pests (Alavanja et al., 2013; Carlock & Dotson, 2010) and together were the most common fumigants identified in the databases used in this study. Metam salts quickly break down in the environment to methyl isothiocyanate (MITC), which is short‐lived in the soil environment because of its volatility (Gadagbui et al., 2014).This volatility of MITC suggests that it can travel beyond the agricultural fields and provides a route for airborne exposure to proximal residential populations. In fact, Woodrow et al. (2018) demonstrated that MITC and its toxic breakdown product, methyl isocyanate (MIC), have been found in quantities that exceed the California EPA’s chronic inhalation reference level in air samples near residences in farming regions in Washington.

Few studies have evaluated the carcinogenic risk to humans from metam exposure. However, Reynolds et al. (2005) found that exposure to metam was linked to a two‐fold increase in the odds ratio for childhood leukemia. Hazard‐adjusted analysis for Yuma County, Arizona, found that among the pesticides evaluated, metam was most closely associated with increased risk of cancer (Sugeng et al., 2013). One nonpeer‐reviewed study of the carcinogenic effect of metam exposure in rodents suggests that rodent exposure to metam induces the development of angiosarcomas (USEPA, 1995). For these reasons, the USEPA, therefore, classifies metam as a “probable human carcinogen.”

We queried the Tox21 database for metam, which showed little evidence for a molecular pathway that could be linked to carcinogenicity (Comptox, 2020b) but when we used MITC, in the query, several assays demonstrate activity influencing the cell cycle, nuclear receptor interactions, and DNA binding (Comptox, 2020a), suggesting that MITC interferes with molecular pathways and cellular physiology that may be linked to potential carcinogenic properties. These outcomes, together with the results presented here suggest that further research into the potential carcinogenicity from exposure to metam and its metabolites is warranted.

### 4.5 Limitations

The issues related to current practices that attempt to geolocate hotspots of cancer are well known and documented in the literature (Goovaerts & Jacquez, 2005; Jacquez, 2004; Jacquez & Greiling, 2003). One of the limitations of this study is the lack of temporal continuity between the secondary data sets used to conduct the analysis presented here (2017 pesticide data vs. 2012–2016 5‐year average cancer incidence data). Although not unique to our study, other variables not measured here could potentially explain the observed relationships between pesticide use and rates of all‐cancer and pediatric cancer incidences. For example, our study does not consider individual‐level exposure or incorporate any demographic factors, such as employment in the agricultural sector or race and ethnicity that may elucidate findings presented. In this way, we assume that the use of state‐level data serves as a substitute measure of individual exposure to pesticides (Mills, 1998). With these limitations in mind, the results of this study should be interpreted with caution.

### 4.6 The Utility of Geospatial Delineation of Carcinogenicity: Conclusions and Future Directions

Geospatial methods of analysis are increasingly recognized as an integral tool for the study of cancer prevention and control, reflecting a growing awareness of the geographical and spatial nuances inherent to cancer distributions (Korycinski et al., 2018). The present study contributes to our understanding of the potential linkages between geospatial distributions of pesticide use and cancer incidence. Our results suggest that large‐scale analyses of potential indicators of cancer incidence, such as pesticide exposure, may provide direction for finer‐scaled studies and in‐depth investigations to determine whether specific exposures may be leading to potential negative human health outcomes. This study has facilitated the identification of priority areas in the U.S. West where increased public health attention may be beneficial, in addition to highlighting specific pesticides of interest. This method of analysis may be particularly useful in informing directed research that can address whether there is a causative, as well as correlative, linkage between environmental factors and adverse health outcomes. This approach can influence policy and decision‐making that may be beneficial to the United States and other agricultural entities, including the European Union (E.U.), Brazil, and China (Donley, 2019).

Future research efforts should continue to examine environmental indicators of cancer incidence on varying scales, particularly at the regional, watershed, and national levels. Future studies should also consider the incorporation of toxicity metrics into explorations of potential linkages between cancer and pesticide exposure. In particular, the Toxicology in the 21st Century program (Tox21), a collaborative interagency research effort aimed at improving toxicity assessment methods, may prove to be a useful resource for such research efforts.

## Conflict of Interest

The authors declare no conflicts of interest relevant to this study.

## Supporting information

Supporting Information S1

## Data Availability

Pesticide data for the United States at county and state resolution can be accessed from the United States Geographic Survey Pesticide National Synthesis Project (USGS‐PNSP) at https://water.usgs.gov/nawqa/pnsp/usage/maps/compound_listing.php. At the time of data retrieval, the 2017 pesticide data were not available in the USGS‐PNSP database for California. Therefore, the data for California were directly sourced from the California Department of Pesticide Regulation Pesticide Use Reporting database (CDPR‐PUR) at https://www.cdpr.ca.gov/docs/pur/pur17rep/statewide_ai_2017.htm. Cancer incidence data can be accessed from the National Cancer Institute State Cancer Profiles at https://statecancerprofiles.cancer.gov/incidencerates.
