# Supplementary material for: Investigation of Relationships Between the Geospatial Distribution of Cancer Incidence and Estimated Pesticide Use in the U.S. West
Source: Geohealth. 2022 May 1;6(5):e2021GH000544. doi: 10.1029/2021GH000544 (PMC9121053; doi:10.1029/2021GH000544)
Supplement: Supplementary file 1 — Supporting Information S1 [file GH2-6-e2021GH000544-s001.pdf]

[GeoHealth]

Supporting Information for

Investigation of relationships between the geospatial distribution of cancer incidence and estimated pesticide use in the U.S. West

Naveen Joseph[1], Catherine R. Propper[2], Madeline Goebel[1], Shantel Henry[2], Indrakshi Roy[3], Alan S Kolok[1]

[1] Idaho Water Resources Research Institute, University of Idaho, Moscow 83843, USA

[2] Department of Biological Sciences, Northern Arizona University, Flagstaff, 86011, USA

[3] Center for Health Equity Research, Northern Arizona University, Flagstaff, 86011, USA

* Corresponding author: Alan S. Kolok (akolok@uidaho.edu)

Contents of this file

Figures S1 to S6

Table S1

1. Supporting Information

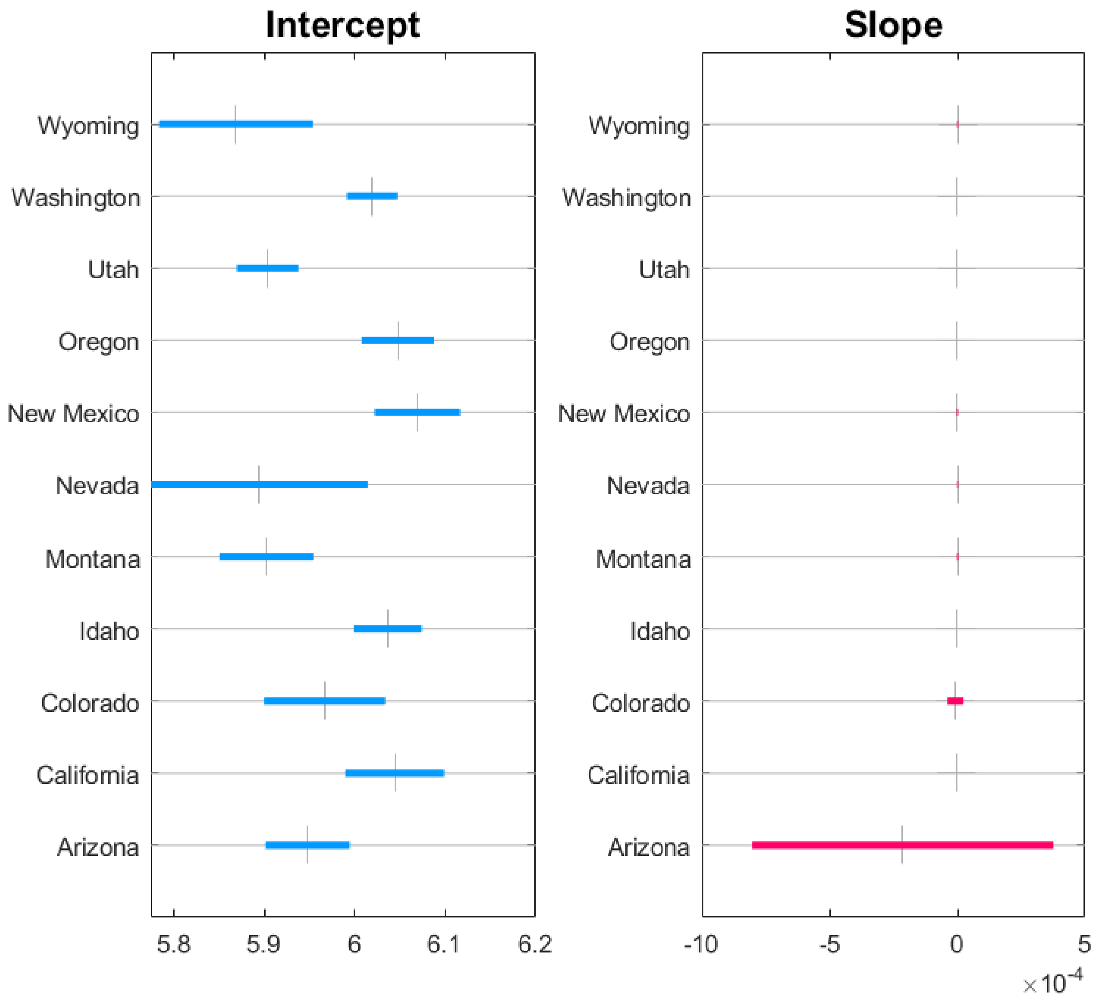

Figure S1. Confidence interval of intercept and slope when grouped by state

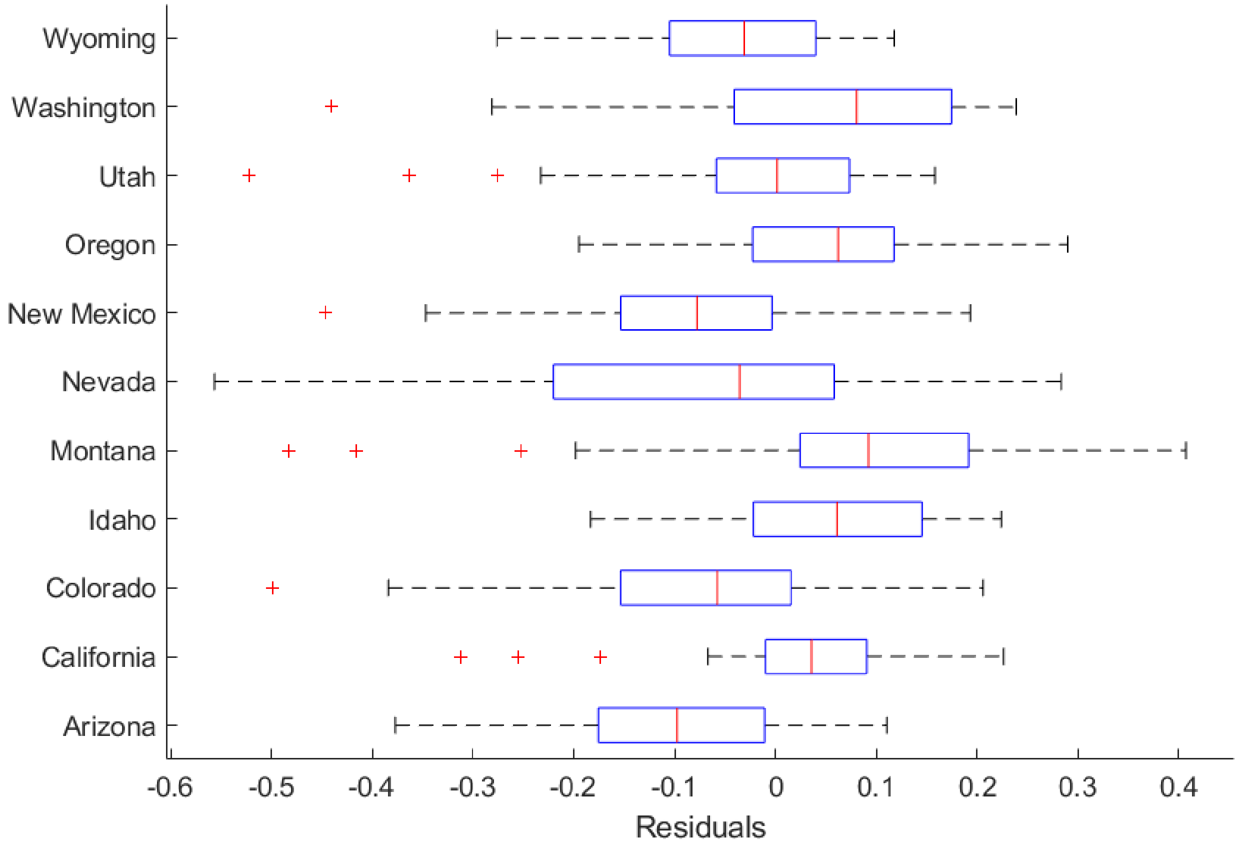

Figure S2. Residuals of linear model for each state

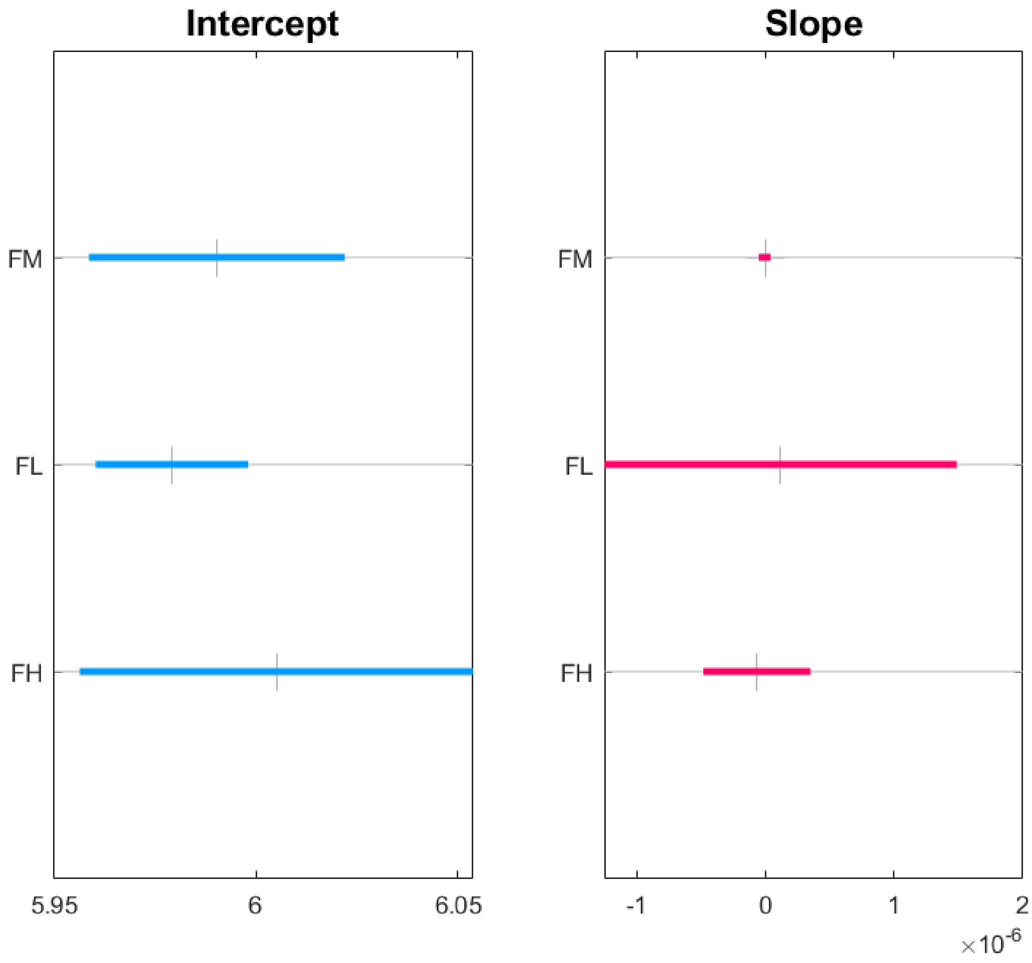

Figure S3. Confidence interval of intercept and slope when grouped by fumigant classes

Table S1. Type-3 tests of fixed effects

Term	F-Stat	*P*-value
Intercept	8079.9	4.47E-69
Fumigant Mass	8.523	0.004836
Fumigant Classes	12.06	3.59E-05

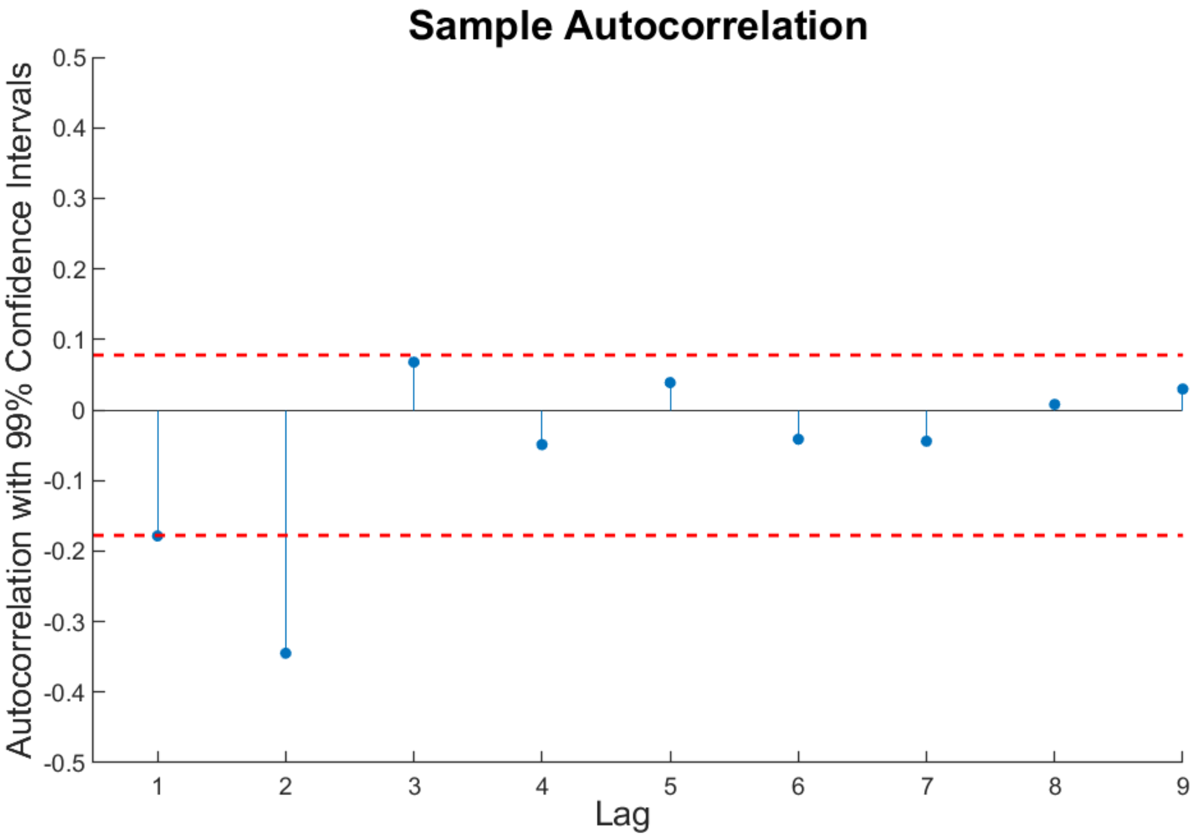

Figure S4. Sample Autocorrelation of residuals from the linear model with 99% Confidence Intervals.

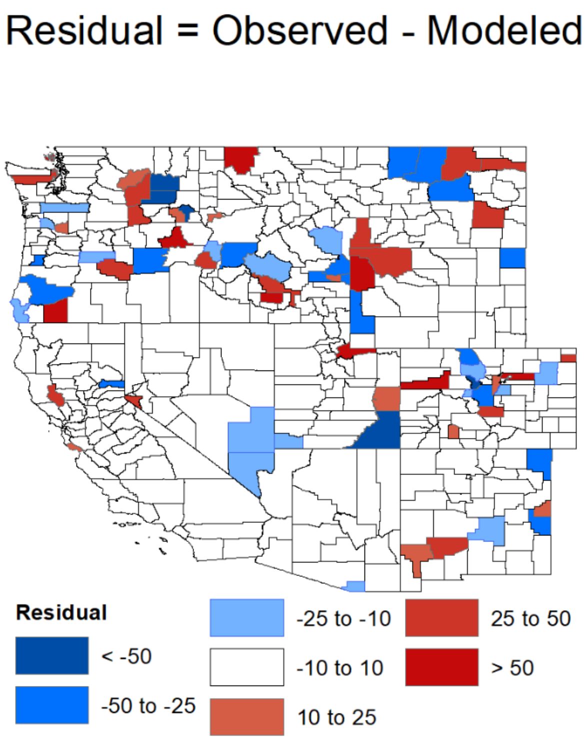

Figure S5. Spatial map of residuals from multilevel model at county scale. The blue color indicates negative residual, red color indicates positive residual, and white color indicates close to zero residual.

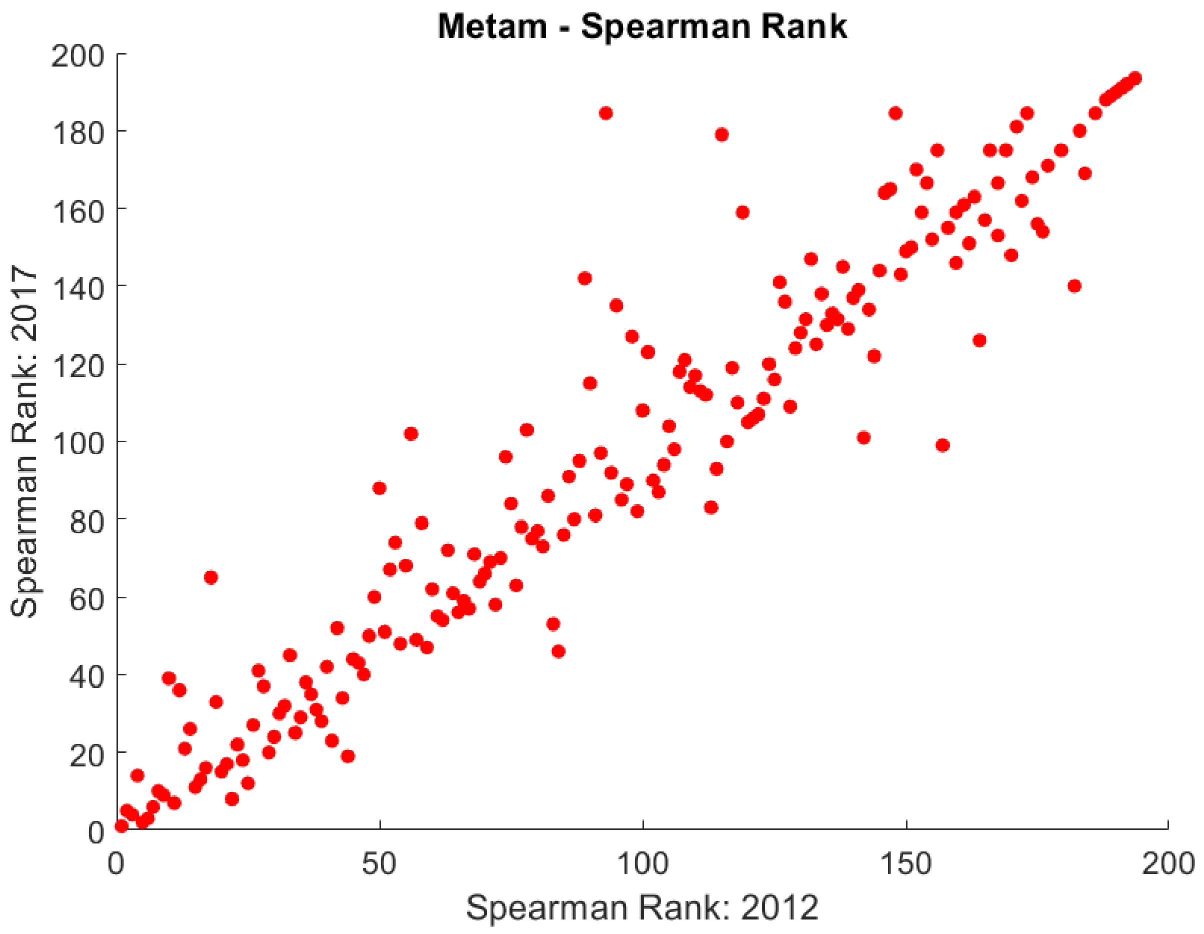

Figure S6. Spearman Rank of Metam for the year 2017 vs Spearman Rank of Metam for the year 2012.
